# Using deep learning to detect upper limb compensation in individuals post-stroke using consumer-grade webcams—A feasibility study

**DOI:** 10.3389/fmed.2025.1645369

**Published:** 2025-11-14

**Authors:** Tim Unger, Benjamin Kühnis, Lena Sauerzopf, Martina R. Spiess, Alexander de Spindler, Andreas R. Luft, Chris Easthope Awai, Josef G. Schönhammer, Elena Gavagnin

**Affiliations:** 1 Data Analytics and Rehabilitation Technology (DART), Lake Lucerne Institute, Vitznau, Switzerland; 2 ZHAW School of Management and Law, Institute of Business Information Technology, Winterthur, Switzerland; 3 ZHAW School of Health Sciences, Institute of Occupational Therapy, Winterthur, Switzerland; 4 Faculty of Medicine, University of Zurich, Zurich, Switzerland; 5 Division of Vascular Neurology and Neurorehabilitation, Department of Neurology and Clinical Neuroscience Center, University of Zurich and University Hospital Zurich, Zurich, Switzerland; 6 Cereneo, Center for Neurology and Rehabilitation, Vitznau, Switzerland; 7 Neurocore Lab, Lake Lucerne Institute, Vitznau, Switzerland; 8 ZHAW School of Engineering, Centre for Artificial Intelligence, Winterthur, Switzerland

**Keywords:** stroke, assessments, movement quality, upper limb, artificial intelligence, computer vision, human pose estimation, webcam

## Abstract

As societies age, the number of individuals experiencing stroke increases, necessitating more effective rehabilitation strategies. Over half of stroke survivors suffer from upper limb impairments, making assessments of sensory-motor function crucial for both improving interventions and tracking progress. Ideally, such assessments could also be performed at home without requiring a therapist's presence. Advances in computer vision and human pose estimation allow for human movement analysis using consumer-grade cameras. This study investigates whether a single webcam, combined with human pose estimation and deep learning algorithms, can automatically detect compensatory movements in persons with stroke performing a drinking task. Twenty participants with stroke with mild to moderate upper limb impairment were recruited. Each participant performed multiple repetitions of the drinking task while being recorded by multiple cameras and an optical motion capture system (OMC) for kinematic ground truth. The videos were labeled by therapists to indicate the presence or absence of compensatory movements. Human poses were extracted from the videos using MediaPipe, and deep learning models were trained to predict these compensatory movements based on MediaPipe keypoints. Several factors affecting compensation detection accuracy were evaluated. Models trained on raw MediaPipe keypoints for inter-person compensation detection failed to generalize, achieving accuracy around 50%. Using custom features instead of raw keypoints improved the accuracy to 70%. In contrast, intraperson classification achieved high accuracy, typically exceeding 90%. Using OMC data significantly improved classification accuracy compared to using MediaPipe keypoints. Camera angle had an effect on accuracy, and convolutional neural networks outperformed long short-term memory networks. Generalizing models remain limited by (1) the measurement uncertainty of human pose estimation and (2) insufficient data representing the full spectrum of compensatory strategies (3) accurate compensation labels. The results demonstrate that deep learning approaches can differentiate between compensatory and non-compensatory movements when movement representations are sufficiently accurate. Future work should improve pose estimation and expand labeled datasets to better reflect the stroke population. While general models are limited in accuracy, personalized models using consumer cameras can support home-based rehabilitation. This digitalized assessment approach has the potential to quantify recovery progress throughout the continuum of care.

## 1 Introduction

The global population is aging rapidly (1). Age is a significant, non-modifiable risk factor for stroke, and the number of stroke cases is expected to rise accordingly (2). Currently, there are over 100 million stroke survivors worldwide, with 12 million new cases reported each year (3). Among these survivors, up to 50% experience chronic upper limb impairments (4), such as reduced range of motion pathological synergies, motor coordination issues and spasticity (5), significantly limiting their independence in daily life. This increasing burden presents major challenges for healthcare systems, emphasizing the urgent need for more effective and scalable rehabilitation strategies to improve patient outcomes.

To address these challenges, maximizing the effectiveness of rehabilitation across inpatient, outpatient, and home-based care is crucial. Tele-rehabilitation and technology-assisted solutions are emerging as essential tools in this effort (6). True recovery of upper limb function in people with stroke through neuroplasticity is the ideal goal, involving the rewiring of neural pathways to restore motor function (7). This forms part of rehabilitation strategies, combining traditional and innovative therapies to restore function (8). However, many people with stroke rely on compensatory strategies, such as using unaffected limbs or adopting maladaptive movement patterns, to regain functionality. While these strategies provide short-term benefits, they may impede neuroplastic recovery and reinforce undesirable movement habits (9). As such, heavy reliance on compensation can serve as a biomarker for limited neuroplastic recovery (10–12).

Common compensatory patterns include trunk leaning, shoulder abduction, and shoulder hiking. To differentiate these from true recovery without increasing therapists workload, there is growing support for adopting instrumented, technology-based assessments (9).

This approach faces three main challenges: selecting an appropriate motor task, accurately measuring movements, and quantifying movement quality/detecting compensatory movements. The drinking task (13), proposed as a standardized motor task by Kwakkel et al. (9), is particularly suitable for stroke rehabilitation. It is a key activity of daily living that is important to achieve autonomy and involves essential movement primitives such as reaching and hand-to-mouth motion, making it both functionally relevant and easy to standardize. In extension of this framework, there are also ongoing approaches to segment specific types of functional movements from continuous activity streams, leading toward continuous evaluation of upper limb movement quality during daily life activities (14).

While optical motion capture (OMC) systems provide highly accurate kinematic data (13, 15), their cost and complexity limit widespread use in clinical and home settings. Alternative approaches, such as RGB- and RGB-D cameras (16, 17), inertial measurement units (IMUs) (18–20), and multi-camera 3D reconstruction systems (21), offer more accessible and affordable solutions but come with trade-offs in accuracy.

Consumer-grade devices with integrated cameras, such as smartphones, tablets, and laptops, are widely accessible and could enable an objective, large-scale remote assessment of movement quality and recovery progress.

**Primary goal:** Evaluate the feasibility of using a single RGB camera and deep learning models to detect compensatory upper limb movements in people with stroke performing the drinking task.

**Secondary goal:** Systematically investigating factors that influence detection accuracy, identifying main challenges that need to be addressed to enable full clinical functionality. To this end, the contribution of the following factors to compensation detection performance is assessed:

1. **Severity of compensation:** Participants were categorized as *mild* or *moderate* compensators based on therapist labeling. Hypothesis: more severe compensations are easier to detect, leading to higher classification accuracy for this subgroup.
2. **Inter- vs. intra-participant classification:** Given the variability in compensation strategies and movement patterns, and the lack of large-scale datasets to fully capture post-stroke compensations, the following factors are assessed:
  - *Inter-Participant*: training on one group of participants and testing on previously unseen participants.
  - *Intra-Participant*: training on a subset of trials from a participant and testing on other trials from the same participant.
3. **Camera perspective and quality of motion data:** As pose estimation accuracy depends on viewpoint, three “at home” reproducible perspectives for the drinking task are compared: frontal view, 45° ipsilateral (close), and 45° contralateral (far). MediaPipe was chosen since it can run on consumer-grade devices, bypassing potential data security issues when processing in the cloud. To isolate the impact of pose estimation noise vs. classification performance, results are bench-marked using:
  - High-quality optical motion capture (OMC) data (gold standard).
  - Noisier human pose estimation (HPE) data from a single RGB camera.
4. **Input features:** Compare custom kinematic features with raw keypoint time-series data for classifying compensatory movements.
5. **Classifier architecture:** Evaluate different classification models:
  - CNN and LSTM for time-series data.
  - Random Forest for custom kinematic features.

This study aims to advance stroke rehabilitation by providing scalable, data-driven tools to quantify movement quality, aligning with European stroke rehabilitation guidelines (9, 11).

## 2 Method and analysis

### 2.1 Participants

The research was conducted in accordance with ethical standards (Declaration of Helsinki an Swiss national standards) as approved by the local ethics committee (BASEC-No: 2022-00491). Participants were recruited from the University Hospital Zurich Stroke Registry and the cereneo clinic. Those eligible for the study were invited to participate in a single measurement session lasting approximately two to three hours. Inclusion criteria required participants to be at least 18 years of age, capable of providing informed consent, and to have a confirmed hemiparetic stroke diagnosis. Additionally, participants needed to have at least partial ability to perform a reaching movement and grasp a cup using a cylindrical grip with the affected hand, without assistance. Exclusion criteria included pre-existing upper limb deficits, such as orthopedic impairments, and other neurological conditions.

Demographic characteristics of the participants were collected and the severity of upper limb sensorimotor impairment was assessed using the Fugl-Meyer Assessment for Upper Extremity (FMA-UE). The FMA-UE was conducted by a trained evaluator along with the measurements of the drinking task during a single session. The final sample involved 20 people with stroke [14 chronic (time since stroke > 6 months), 6 subacute (time since stroke between 1 to 6 months)], with a mean FMA-UE score of 51.7 (11.5) assessed on the day of recording, reflecting a moderate level of upper limb impairment and matching demographic data (mean age 72, 14 males, and 6 females) with previous studies (22). Participants in the subacute phase were recruited during their rehabilitation stay at the cereneo clinic.

### 2.2 Measurement procedure

**Figure 1:**
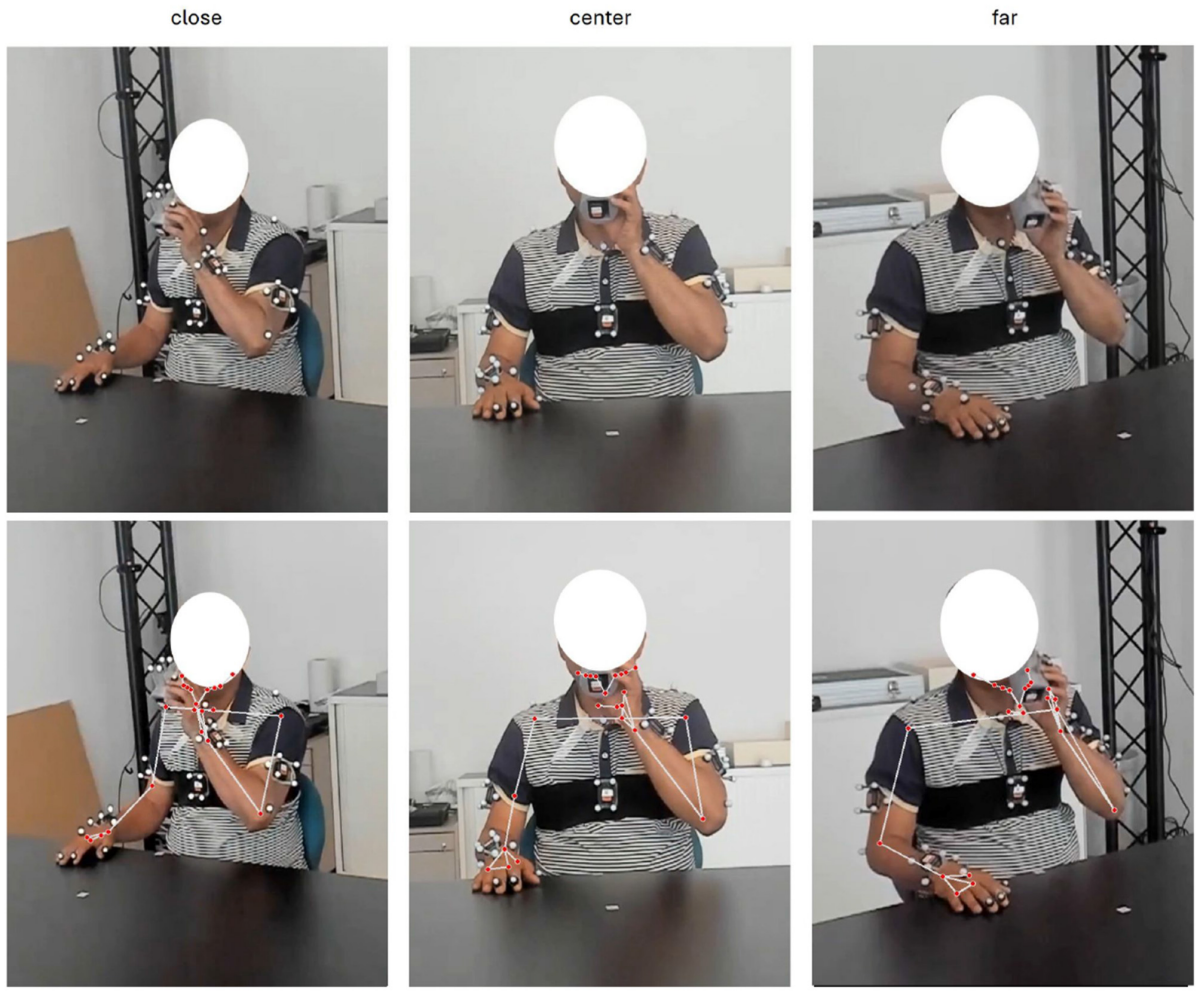
Participant performing drinking task, recorded from three different camera perspectives (“close,” “center,” “far”) and with optical motion capture and corresponding keypoints overlay.

The measurement procedure, which followed established protocols from previous research (22), required participants to begin and end a drinking task in a standardized pose. The cup was consistently placed 30 cm from the table's edge (see Figure 1). It was filled with approximately 100 ml of water and refilled as needed between trials. Each repetition of the drinking task was considered a trial. Participants were instructed to take a sip of water during each trial. In the rare instances where participants dropped the cup and spilled water (as occurred with 2 participants), the water was replaced with a ball of similar weight, and participants were instructed to continue with the drinking movement regardless. Each participant completed 40 trials of the drinking task with their less affected arm, followed by 40 trials with their more affected arm. The less affected arm served as a control, representing proxy data for able-bodied, age-matched individuals. Every trial was recorded individually, participants received verbal instructions at the start of each trial, and recording was halted upon trial completion and restarted at a new trial. After several repetitions, participants were asked whether they required a break; if requested, a break was provided to minimize potential fatigue effects. Additional trials were conducted if any were deemed invalid (e.g., movement beginning before recording or incomplete movement). After completing the drinking task, the FMA-UE was performed.

### 2.3 Measurement systems

This study employed two primary measurement systems: webcams and an OMC system. The measurement setup, including the table, cup, recording perspectives, and OMC markers on the participant, is illustrated in Figure 1. Both systems were synchronized automatically using an LED triggered by the OMC (master), which was visible in the webcam field of view to mark the start and end of recording.

*OMC:* The OMC system operated at a sampling frequency of 100 Hz, utilizing technologies from Vicon, Qualisys, or Optitrack, depending on the specific measurement setup, leveraging 7 to 10 OMC cameras. Nine retroreflective markers positioned on anatomical landmarks of the arm, trunk, and face were used for they analysis, in accordance with the standardized marker setup of previous studies that measured the Drinking Task using OMC (13, 22). The cup used in the task was 3D printed and allowed the attachment of OMC markers, relevant for phase classification.

*Webcams:* Three Logitech Brio 4k webcams were positioned in front of the participant at a distance of approximately 1.5m. One camera was placed at a 0-degree recording angle (center camera), while the other two were placed at 45-degree recording angles toward the frontal plane of the participant. The 45-degree camera referred to as “close” was positioned on the side where the drinking task was performed. The webcams were internally synchronized and continuously recorded at 60 frames per second with a resolution of 1,080p (1,920 × 1,080). The choice of camera perspectives was based on their reproducibility in clinical or home settings using tablets, laptops, or comparable end-user devices, with the goal of identifying the optimal recording angle for classification accuracy.

### 2.4 Data preparation and modeling

Videos from each camera were automatically segmented into trials using LED signals synchronized with the OMC system. OMC data further enabled segmentation of the drinking task into five phases, following Alt Murphy (22): (1) reaching (including grasping), (2) forward transport (glass to mouth), (3) drinking, (4) back transport (glass to table, including release), and (5) returning (hand to initial position).

Experienced therapists labeled each phase for compensatory movements based on the checklist by Barth et al. (23) and the Reaching Performance Scale (24) and described in full detail in Sauerzopf et al. (25). Shoulder compensations were defined as excessive scapular elevation, shoulder hiking, or abduction in cases of insufficient shoulder flexion. Hand compensations included inadequate hand opening or grasping. Approximately 40 trials per participant were labeled for the affected side, while less-affected side trials were consistently labeled as *no compensation*. This labeling provided the ground truth across participants, phases, and compensation types. Due to measurement noise, reliable hand compensation detection was not feasible with MediaPipe, and subsequent analyses therefore focused on shoulder compensation.

Compensation severity was categorized as *mild* (≥1 phase labeled *yes*), *moderate* (≥3 phases labeled *yes*), and *less-affected* (all phases *no*). Participants without compensation (no trial labeled as compensation “yes”) were excluded resulting in 13 participants with shoulder compensation. Undersampling was used for equal representation of compensatory and non-compensatory trials. Data normalization included scaling by ear-to-ear distance and mirroring of right-hand trials to ensure consistent left-hand representation.

Pose data were extracted from all videos using MediaPipe BlazePose (heavy model) (26). From the 33 available 3D keypoints, only upper-body keypoints (0–22) were retained, and analyses used 2D coordinates due to unreliable depth information in seated participants. Three camera perspectives (close, far, center) were processed separately to allow subsequent evaluation of viewpoint effects.

In addition to raw keypoints, custom kinematic features, following Alt Murphy (22), were derived from MediaPipe and OMC (Table 1). These included measures such as minimum elbow extension, maximum shoulder angle, and peak tangential velocity. Certain features required phase durations, which were computed both from OMC and directly from MediaPipe keypoints. This dual approach allowed comparisons between precise OMC-based phase segmentation and noisier, purely vision-based segmentation. OMC-derived features were assumed to have minimal measurement error and therefore served as a benchmark. A Random Forest classifier (20 estimators) was trained on these features.

**Table 1:** Custom features based on keypoints.

**Name**	**Description**
Min Elbow Extension	Minimum elbow extension (angle of keypoints 11, 13, 15) during the reaching phase.
Average Smoothness	Calculates the average smoothness of the movement based on the variance in velocities between the shoulder, elbow, and hand keypoints of the reaching arm.
Number of Movement Units	Number of movement units measured during the phases.
Peak Average Velocity	Peak elbow velocity (pixel/frame) during the reaching phase. A sliding window of 8 is applied to smooth the data.
Peak Tangential Velocity	Peak tangential velocity (pixel/frame) during the reaching phase. Calculated as the distance between the shoulder (keypoint 11) and hand (keypoint 15) per frame. A sliding window of 8 is applied to smooth the data.
Max Shoulder Angle	Max shoulder angle (keypoints 11, 13, and 23) over all phases.

For raw keypoints, two deep learning models were tested: a 1D Convolutional Neural Network (CNN) with three convolutional layers (64 filters), global average pooling, and a softmax classifier; and a Long Short-Term Memory (LSTM) network with two recurrent layers (50 units each), dropout (0.2), a dense layer (20 units), and a softmax output. Both CNN and LSTM were trained with the Adam optimizer and employed sparse categorical cross-entropy as the loss function, and used a batch size of 32. To mitigate overfitting and promote efficient training, early stopping was employed. Parameters were chosen through preliminary searches to balance performance and complexity. For custom features, a Random Forest served as a classical baseline.

All analyses were implemented in Python (3.9.6). Video processing used OpenCV (opencv-python==4.8.1.78); deep learning used TensorFlow (tensorflow==2.15.0); and Random Forest classification and utilities used Scikit-learn (scikit-learn==1.4.0).

### 2.5 Comparison of key factors affecting compensation detection performance

**Figure 2:**
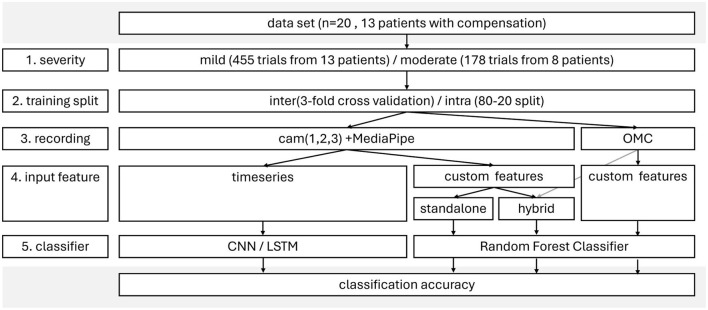
FlowChart of analysis.

Multiple analysis pipelines were compared and five key factors were systematically varied, assessing each factors impact on classification accuracy (ref Figure 2). The performance of each pipeline was determined by its test accuracy, recall, and precision in detecting compensations. The test accuracy was defined by the percentage of trials correctly classified as compensation *yes* or *no*.

1. **Severity of compensation:** The impact of compensation severity was assessed by splitting the dataset into data containing mild and moderate compensations and data with only moderate compensations. This allowed us to determine whether the pose estimation method could detect subtle compensations or was limited to larger, more pronounced movements.
2. **Inter- vs. intra-participant classification:** Two training and validation strategies were applied. Intra-participant classification involved splitting trials from the same participant into training and testing sets to evaluate performance within a single individual (80–20 training-test split of trials). For inter-participant classification, the dataset was partitioned by participants to evaluate the model's ability to generalize to unseen individuals with varying compensation patterns. Stratified group 3-fold cross-validation (StratifiedGroupKFold) was applied, ensuring that all data from a given participant was confined to either the training or the test set within each fold. This approach preserved class balance across folds while preventing information leakage between participants.
3. **Camera perspectives and quality of motion data:** Three camera angles (far, close, and center - see Figure 1) were tested to evaluate the influence of perspective on pose estimation accuracy and, consequently, on the classification of compensatory movements. The selected angles were chosen to reflect feasible setups in clinical and home settings.
  High quality motion data from OMC, combined with the movement quality measures from Murphy et al. (22), served as a baseline condition to disentangle the measurement uncertainty of MediaPipe from the inherent uncertainty in compensatory movement classification. Three data modalities were tested with increasing data quality (MediaPipe standalone: features and phase classification based on MediaPipe keypoints; Hybrid: features based on MediaPipe and phase classification based on OMC; OMC: features and phase classification based on OMC). A Random Forest classifier was used as a robust classical model to benchmark deep learning approaches.
4. **Input features:** Two types of input features were tested: first, custom kinematic features derived from kinematics and movement phases (see Table 1) and second, timeseries-data based on MediaPipe keypoints.
5. **Classifier architectures:** For raw keypoint inputs, 1D Convolutional Neural Networks (CNNs) and Long Short-Term Memory networks (LSTMs) were tested as standard methods for time-series data classification problems. For kinematic feature inputs, a Random Forests was used as a classical machine learning baseline, allowing for a comparison of deep learning against traditional approaches.

## 3 Results

The analysis yielded classification accuracies across a range of experimental settings, varying by compensation severity (mild vs. moderate), training split (inter- vs. intra-participant), camera perspective (close, center, far), and motion data quality, input features and classifier architecture. Three data modalities were compared: (1) standalone MediaPipe keypoints, (2) a hybrid approach combining MediaPipe keypoints with OMC-based phase classification, and (3) complete OMC data as a high-fidelity baseline.

Results (test accuracy, recall and precision) are summarized in Table 2 for custom features, and Table 3 for time-series data (intra-participant, CNN). The findings are structured according to the key factors hypothesized to influence the classification performance.

1. **Severity of compensation**
  The severity of compensatory movements influenced classification performance differently across evaluation settings (Table 2). In inter-participant scenarios, mild compensations were classified more accurately and with higher recall than moderate compensations. For example, in the Hybrid–close camera condition, inter-participant accuracy was 0.70 (± 0.02) for mild compensations compared to 0.58 (± 0.09) for moderate, and recall was 0.64 (± 0.05) (mild) vs. 0.30 (± 0.17) (moderate). Precision, however, was slightly higher for moderate compensations (0.76 ± 0.22) compared to mild (0.72 ± 0.03), although with greater variability. These results suggest that mild compensations, while more subtle, were generally more consistently detected across participants, whereas moderate compensations led to less reliable recall.
  In intra-participant classification, the differences were less pronounced, and in some cases, moderate compensations slightly outperformed mild ones. For instance, accuracy values in the close camera condition were 0.88 (mild) and 0.91 (moderate), while precision improved from 0.72 (mild) to 0.76 (moderate). This indicates that when training and testing on the same individual, classification performance remained high regardless of compensation severity, with moderate compensations offering a small advantage.
  Using keypoint time-series data with CNNs, both mild and moderate compensation groups achieved consistently high intra-participant accuracies (0.92–0.95; Table 3).
  In addition to stratified 3-fold cross-validation with undersampling, a stricter leave-one-subject-out (LOSO) analysis with SMOTE (see Supplementary material) was also performed. The results were highly consistent across both evaluation schemes for OMC mild and for the hybrid mild/moderate conditions, supporting the robustness of these findings. The only notable deviation was in OMC Moderate, where LOSO performance degraded compared to stratified CV, underscoring the difficulty of generalizing this condition with limited sample size.
2. **Inter- vs. intra-participant classification**
  A pronounced performance gap was observed between inter- and intra-participant evaluations. Intra-participant models consistently achieved substantially higher accuracy, recall, and precision across all feature sets and camera perspectives. For instance, using custom features with Standalone-center for mild compensations, accuracy improved from 0.48 (± 0.10) in the inter-participant evaluation to 0.83 in the intra-participant split. A similar effect was observed for precision (0.49 ± 0.14 inter vs. 0.83 intra) and recall (0.32 ± 0.07 inter vs. 0.83 intra). The same pattern was evident with custom features from higher-quality data: in OMC-based classification, inter-participant accuracies ranged between 0.65 (± 0.22) (moderate, inter) and 0.71 (± 0.06) (mild, inter), while intra-participant accuracies were consistently high at 0.92–0.94. These findings indicate that generalization across participants is substantially more challenging than within-participant classification, highlighting the strong individual variability in compensatory movement patterns.
  In contrast, keypoint time-series models failed to generalize for inter-participant settings (accuracy around 0.5), but performed well in intra-participant classification (0.92–0.95).
3. **Camera perspective and quality of motion data**
  Both camera perspective and data quality strongly influenced classification outcomes. As expected, the OMC condition consistently provided the highest performance, with inter-participant accuracies of 0.71 (± 0.06) for mild compensations and to 0.65 (± 0.22) for moderate compensations, and near-perfect intra-participant accuracies 0.92 (mild) and 0.93 (moderate). Hybrid features yielded similar performance to Standalone, with accuracy values that were the same or up to 0.07 higher under otherwise comparable conditions. For example, in the mild–close condition, Hybrid improved inter-participant accuracy from 0.63 (± 0.07) to 0.70 (± 0.02), and intra-participant accuracy from 0.83 to 0.88. Camera perspective also played a role: the close perspective outperformed both center and far perspectives, particularly for moderate compensations. In the Hybrid–close condition, inter-participant accuracy (mild) reached 0.70 (± 0.02), compared to 0.48 (± 0.12) for the center view and 0.58 (± 0.09) for the far view. Notably, the center–moderate inter-participant condition performed extremely poorly, with accuracies ranging only from 0.42–0.43, recall from 0.01–0.02, and precision from 0.05–0.11 across Standalone and Hybrid models, indicating a near-complete failure to detect compensations in this setting. Intra-participant accuracies were also highest in the close (moderate) condition (0.91) compared to 0.85–0.88 in far and center views. These results confirm that richer kinematic detail (OMC or closer viewpoints) substantially improves classification performance, while distant perspectives degrade performance, especially in inter-participant classification.
4. **Input features**
  The choice of input features had a marked impact on performance, but this effect differed between inter- and intra-participant evaluations. With custom features, both inter- and intra-participant classification were feasible, although inter-participant accuracies remained modest (ranging from 0.48–0.70 for mild compensations and 0.42–0.60 for moderate compensations). In contrast, time-series models (CNN) only converged in the intra-participant setting, where they achieved substantially higher and more consistent performance. Specifically, CNN intra-participant test accuracies were between 0.92 and 0.95 across all conditions, with balanced precision and recall values (≥ 0.92). For example, in the mild–far condition, intra-participant test accuracy increased from 0.88 (custom features) to 0.94 (CNN time-series). Similarly, in the moderate–close condition, accuracy improved from 0.91 (custom features) to 0.95 (CNN).
  By contrast, in the inter-participant evaluation, time-series classification failed to converge and yielded accuracies close to or below 0.50, highlighting struggle to generalize across participants.
5. **Classifier architecture**
  When comparing model architectures for time-series classification, CNNs consistently outperformed LSTMs. In the intra-participant setting, CNNs achieved high and balanced performance across all conditions, with test accuracies between 0.92–0.95, while LSTMs failed to converge and produced accuracies close to or below 0.50, effectively at chance level. For instance, in the moderate–far intra condition, CNN performance reached 0.93 accuracy, 0.93 recall, and 0.93 precision, whereas the LSTM failed to surpass random performance. In the inter-participant setting, both CNN and LSTM failed to converge, indicating that temporal models in their current form were unable to capture inter-individual variability.
  These findings suggest that CNNs are well-suited to intra-participant time-series classification, effectively leveraging keypoint trajectories to detect compensations, while LSTMs were unstable and ineffective in this task. However, the failure of both CNN and LSTM in inter-participant classification emphasizes that temporal models alone may not be sufficient to handle between-participant variability in compensatory movement patterns.

**Table 2:** Test Accuracy (A), Recall (R), and Precision (P) for inter- and intra-participant classification using Standalone, Hybrid, and OMC features.

**Custom features method**			**Standalone**		**Hybrid**		**OMC (A/R/P)**	
**Comp**.	**Cam**.	**Metric**	**Inter**	**Intra**	**Inter**	**Intra**	**Inter**	**Intra**
mild	center	Accuracy	0.48 (± 0.10)	0.83	0.48 (± 0.12)	0.82	0.71 (± 0.06) 0.63 (± 0.14) 0.84 (± 0.18)	0.92 0.92 0.92
		Recall	0.32 (± 0.07)	0.83	0.37 (± 0.12)	0.82		
		Precision	0.49 (± 0.14)	0.83	0.48 (± 0.16)	0.83		
	far	Accuracy	0.57 (± 0.05)	0.86	0.58 (± 0.09)	0.88		
		Recall	0.42 (± 0.06)	0.86	0.48 (± 0.15)	0.88		
		Precision	0.61 (± 0.09)	0.86	0.61 (± 0.10)	0.88		
	close	Accuracy	0.63 (± 0.07)	0.83	0.70 (± 0.02)	0.88		
		Recall	0.62 (± 0.21)	0.83	0.64 (± 0.05)	0.88		
		Precision	0.63 (± 0.08)	0.83	0.72 (± 0.03)	0.88		
moderate	center	Accuracy	0.43 (± 0.03)	0.89	0.42 (± 0.04)	0.85	0.65 (± 0.22) 0.48 (± 0.40) 0.59 (± 0.34)	0.93 0.93 0.94
		Recall	0.01 (± 0.01)	0.89	0.02 (± 0.00)	0.85		
		Precision	0.05 (± 0.04)	0.90	0.11 (± 0.06)	0.86		
	far	Accuracy	0.58 (± 0.06)	0.89	0.60 (± 0.10)	0.88		
		Recall	0.28 (± 0.12)	0.89	0.33 (± 0.16)	0.88		
		Precision	0.68 (± 0.16)	0.89	0.67 (± 0.21)	0.88		
	close	Accuracy	0.55 (± 0.09)	0.93	0.58 (± 0.09)	0.91		
		Recall	0.29 (± 0.20)	0.93	0.30 (± 0.17)	0.91		
		Precision	0.70 (± 0.25)	0.93	0.76 (± 0.22)	0.91		

Rows are grouped by compensation severity and camera angle. Results are shown as mean (± SD) for inter-participant and single-point estimates for intra-participant evaluation.

**Table 3:** Intra-participant comparison of CNN Models using MediaPipe keypoints time-series data with different compensation intensity subgroups (mild, moderate) and camera angles (far, close, center).

**Compensation**	**Camera**	**Train**			**Test**		
		**Accuracy**	**Recall**	**Precision**	**Accuracy**	**Recall**	**Precision**
Mild	Center	0.94	0.94	0.94	0.94	0.94	0.94
Mild	Far	0.92	0.92	0.93	0.94	0.94	0.95
Mild	Close	0.94	0.94	0.94	0.92	0.92	0.92
Moderate	Center	0.96	0.96	0.96	0.92	0.92	0.92
Moderate	Far	0.97	0.97	0.97	0.93	0.93	0.93
Moderate	Close	0.95	0.95	0.95	0.95	0.95	0.95

Compensation severity was categorized as mild if at least one phase was labeled as displaying compensation, moderate if this was the case for three or more phases.

## 4 Discussion

Recent advances in computer vision based 2D pose estimation using single RGB cameras present a promising low-cost alternative for movement analysis. While 2D pose estimation has achieved high accuracy levels, its performance is highly dependent on recording conditions such as camera angle and the complexity of the movement. Errors in joint angle estimation can range from under 10 degrees to over 50 degrees, highlighting the need to optimize recording setups for specific applications. In the context of stroke rehabilitation, identifying the optimal camera perspective, pose estimation algorithm, and outcome measures for detecting upper limb compensations during the drinking task remains a significant challenge.

Although pose estimation algorithms are improving, they do not yet match the accuracy of OMC systems, necessitating alternative methods to quantify movement quality. Movement therapists bring invaluable expertise to this task, but their availability is limited, especially in home-based settings. This underscores the need for accessible, automated tools capable of detecting compensations with therapist-level insights. Previous work has demonstrated the feasibility of using consumer-grade webcams and deep learning classifiers to detect compensatory movements during a drinking task (30), but further investigation is needed to enhance the robustness and generalization of these methods.

### 4.1 Summary of findings

This study demonstrates that compensatory movement detection using a single RGB camera is feasible, with several factors significantly influencing classification accuracy. For inter-participant evaluation, mild compensations were detected more reliably than moderate ones, whereas in intra-participant evaluation, moderate compensations were detected more reliably than mild ones. Intra-participant classification yielded consistently high accuracy, while inter-participant classification remained challenging, especially with MediaPipe-based models. The close view camera perspective overall provided better results than frontal and far cam perspectives. Models using custom kinematic features outperformed those using raw keypoints for inter-participant classification, especially when paired with high-quality phase classification from OMC data. Conversely, raw keypoints performed slightly better in intra-participant settings using CNNs. Across all settings, CNNs outperformed LSTMs using time-series data.

### 4.2 Interpretation and relation to previous work

Recent studies on compensation detection in stroke rehabilitation have explored various sensing and analysis methods. According to Wang et al. (27), body-worn sensors and OMC dominate the field, though markerless systems like 2D pose estimation are gaining traction. Machine learning remains underused, and most studies involve small samples, limiting generalizability.

The drinking task has been analyzed using OMC (22), IMUs (28), and markerless approaches (29). While OMC offers high accuracy, it is impractical for home use; IMUs face drift and calibration challenges in real-world settings; and 2D pose estimation, though more accessible, may lack precision. Lin et al. (30) showed promising intra-subject results for detecting compesnation in people with stroke performing the drinking task using BlazePose, but their study lacked inter-participant validation.

The need for objective and reliable labeling remains a challenge. Jose et al. (31) and Sauerzopf et al. (32) highlight inter-rater variability in video-based scoring, emphasizing the need for automated, standardized approaches.

**Figure 3:**
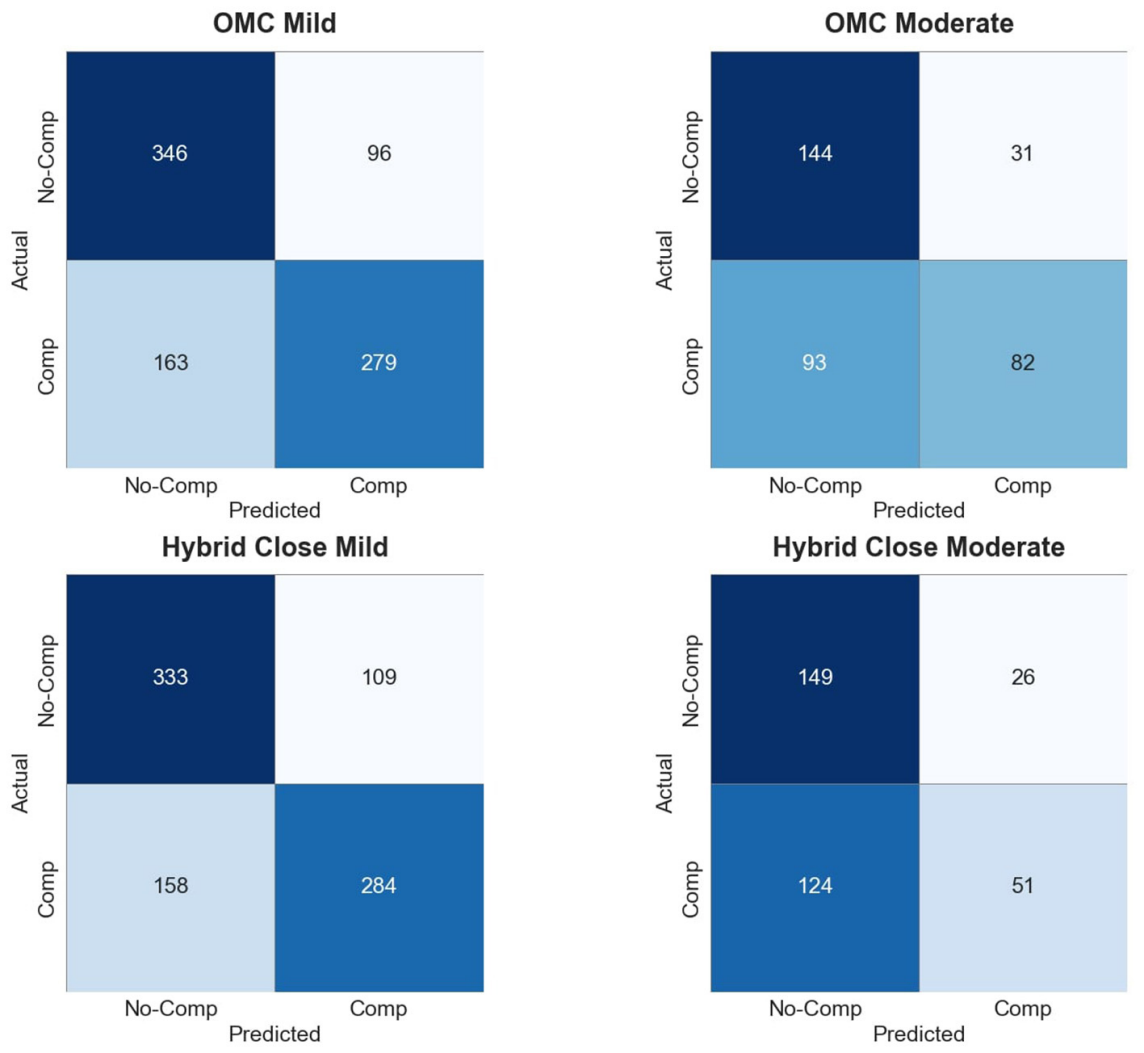
Confusion matrices for OMC and Hybrid Close models under mild and moderate conditions.

Building on these findings, this study uses MediaPipe-based 2D pose estimation with deep learning for automated compensation detection during a drinking task. The findings are discussed around the five factors influencing classification performance:

1. **Severity of compensation**
  Contrary to our initial hypothesis, mild compensations were generally classified more accurately than moderate compensations in inter-participant settings. For example, with OMC features, inter-participant accuracy reached 0.71 (± 0.06) for mild but only 0.65 (± 0.22) for moderate; recall and precision followed the same pattern [mild: 0.63 (± 0.14) recall, 0.84 (± 0.18) precision; moderate: 0.48 (± 0.40) recall, 0.59 (± 0.34) precision]. These findings, together with the confusion matrices (Figure 3), indicate that generalization across participants is more robust for mild compensations. A likely explanation lies in dataset composition: there were substantially more mild samples, whereas only five patients exhibited sufficient moderate compensations, leading to high variance and lower reliability for that group. The confusion matrices (Figure 3) further show that moderate compensations are often misclassified in inter-participant settings, which can be attributed to the small number of moderate cases and the resulting variability across patients. While moderate compensations appear more distinct within individuals (intra-participant), their scarcity makes them harder to generalize reliably across participants.
  The original hypothesis was only supported in intra-participant classification, where moderate compensations achieved slightly higher accuracies (OMC: 0.93 vs. 0.92 for mild), suggesting that more pronounced compensations are easier to detect within individuals. Also, labeling inconsistencies, particularly in edge cases, introduce noise into the data (32) and likely affect test accuracy. This underscores the need for larger and more balanced datasets, as well as more robust labeling and feature representations, to capture both subtle and pronounced compensatory movements with equal reliability.
2. **Inter- vs. intra-participant evaluation**
  As expected, intra-participant models outperformed inter-participant models. This likely reflects the individuality of compensatory strategies, which may not generalize well across subjects. To improve inter-participant compensation detection, expanding the dataset either through broader data collection or augmentation using synthetic movement data could help better represent the variability in post-stroke compensation strategies during the drinking task. The performance gap is especially pronounced with less accurate data sources, emphasizing that inter-participant classification requires movement patterns to remain detectable despite increased measurement noise. This is further highlighted by the improved inter-participant accuracy when using high-fidelity OMC data.
3. **Camera perspective and data quality**
  While MediaPipe is optimized for frontal views, the close camera consistently performed best in this study. This likely reflects the importance of sagittal-plane information for tasks like reaching, which are difficult to capture from a purely frontal perspective. The close camera offered a mixed frontal-sagittal view, improving the ability to detect relevant movement characteristics. In contrast, the moderate–center camera condition underperformed, most likely because certain features were less reliable in the absence of depth information that cannot be captured from a frontal view. Likewise, the mild–far condition was negatively affected by occlusions, as the moving arm was partially obstructed at greater distances, leading to reduced classification performance.
  Higher-quality motion data consistently led to better classification performance. This was evident not only when comparing OMC to MediaPipe but also in the hybrid approach, where MediaPipe keypoints were paired with OMC-derived phase segmentation. These results demonstrate that deep learning models are capable of identifying compensatory patterns provided that movement representations are sufficiently accurate, both in spatial quality and temporal segmentation.
4. **Input features**
  MediaPipe keypoint time-series data failed to generalize for inter-participant classification, likely due to a low signal-to-noise ratio. The limited number of participants and noise in the keypoints likely prevented the models from learning robust patterns of compensation. Improving generalization would require both higher-quality time-series data and a larger training population. Intra-participant classification using MediaPipe time-series data achieved accuracies of 0.92–0.95 which is in line with Lin et al. (30), reporting 0.92.
  The improved performance of intra-participant models using custom features shows the benefit of prior feature selection in enhancing the signal-to-noise ratio. For example, accuracies reached up to 0.70 in the mild group (close camera, hybrid approach), suggesting that meaningful kinematic features can still be extracted even from noisier data sources if domain knowledge is incorporated effectively.
5. **Classifier architecture**
  The CNN outperformed the LSTM by reliably capturing local temporal–spatial patterns in joint trajectories, achieving intra-participant accuracies ≥0.92. In contrast, the LSTM failed to converge (accuracies around 0.5). LSTMs are more data-demanding and sensitive to variability, which may have amplified instability given the limited dataset size. Both models failed in inter-participant classification, indicating that architectural choice alone is insufficient for generalization, and highlighting the need for strategies such as normalization or domain adaptation.

### 4.3 Limitations and future directions

This study demonstrates the potential of consumer-grade webcams and deep learning to detect upper limb compensatory movements in people with stroke during a drinking task. However, several limitations, must be addressed in future research to achieve robust, automated compensation detection and enhance clinical applicability.

The limited sample size, comprising only 20 participants with 13 exhibiting compensatory movements, restricted the dataset's representativeness across diverse compensation patterns. The little amount of data did limit the use of validation data during training process, usually leading to overfitting of the model. This study solely relied on test data set to evaluate the perfomance. Furthermore, the drinking task's motor requirements excluded individuals with severe impairments, skewing the sample toward those with higher functionality. This probably hampered the models' ability to generalize to the broader stroke population. Future research should prioritize larger cohorts spanning a wide range of impairment levels and exploring methods to synthetically enrich the data set with data augmentation methods (33–35) as previously successfully implemented in optimizing rare disease gait classification (36). Using the unaffected side of participants as a proxy for able-bodied movements may represent a limitation, even when therapists label these movements as non-compensatory. Future work should therefore include movement data from age-matched able-bodied participants for comparison.

Pose estimation accuracy was a significant challenge, as MediaPipe's 2D keypoints probably struggled to discern subtle differences between compensatory and non-compensatory movements.

Using only MediaPipe was an explicit choice from the outset, as it can run efficiently on consumer-grade devices and avoids data security concerns associated with cloud-based processing. However, this lightweight algorithm comes at the cost of reduced accuracy, which represents a key limitation of the present study. Future work should therefore include comparative analyses with more advanced pose estimation models to quantify potential performance gains and evaluate whether the trade-off between accessibility and accuracy can be optimized.

This study was conducted in a controlled setting with standardized conditions, including fixed camera positions and only one person in view. Real-world applications, however, may introduce additional challenges such as multiple people in frame, varying lighting conditions, diverse camera devices, and challenges in how the application is used without support. Future work should examine these factors and their impact in real-life scenarios, and investigate how applications on tablets, smartphones, or laptops can be designed to maximize usability for people with stroke, including clear user guidelines and tutorials and potential fine-tuning options of the classification model leveraging movement data of the specific end user.

The binary labeling system for compensatory movements, while practical, lacked granularity for detailed clinical insights and was susceptible to inter- and intra-rater variability (32). Manual labeling by therapists was labor-intensive and might have introduced noise into the training data. Inconsistent annotations obscure compensatory patterns, especially in small datasets, undermining model performance. As described in full detail in Sauerzopf et al. (32), inter-rater reliability was good for reaching, drinking, and returning phases (ICCs 0.75–0.88) and moderate for transport phases (ICCs 0.65–0.68). Future work should develop data-driven labeling approaches, such as unsupervised learning or continuous assessment scales, to improve consistency and reduce dependence on manual annotations.

In summary, advancing tracking precision, expanding dataset diversity, and refining labeling methods are critical to developing low-cost, user-friendly tools for stroke rehabilitation. These improvements will enhance patient outcomes in clinical and home settings, aligning with the vision of scalable, technology-driven care.

## 5 Conclusion

Enhancing the effectiveness of rehabilitation strategies and home therapy, along with the implementation of smart home assessments, will be essential in the future of patient care. For these technologies to be successfully adopted, they must be low-cost, accessible, and user-friendly. This study demonstrates that with tailored algorithms, current computer vision technologies can already be applied to detect compensatory movements. However, the importance of accurate movement representation and the need for larger datasets remain critical for developing models that generalize well. Continued progress in technology and data collection methods will further enhance the potential of computer vision in rehabilitation contexts, ultimately contributing to more effective and accessible patient care.

## Data Availability

The datasets presented in this article are not readily available because anonymized data set will be open sourced in the future. Requests to access the datasets should be directed to tim.unger@llui.org.
