# Supplementary material for: Using deep learning to detect upper limb compensation in individuals post-stroke using consumer-grade webcams—A feasibility study
Source: Front Med (Lausanne). 2025 Nov 14;12:1645369. doi: 10.3389/fmed.2025.1645369 (PMC12660197; doi:10.3389/fmed.2025.1645369)
Supplement: Supplementary file 1 [file Data_Sheet_1.pdf]

Using Deep Learning to Detect Upper Limb Compensation in Individuals Post-Stroke Using Consumer-Grade Webcams - A Feasibility Study

T. Unger [1†*], B. Kühnis [2†], L. Sauerzopf [3,4], M.R. Spiess [3,4], A. de Spindler [2], A. Luft [5,6,8], C. Easthope Awai [1], J.G. Schönhammer [5,8‡], E. Gavagnin [2,7‡]

[1] *Data Analytics & Rehabilitation Technology (DART), Lake Lucerne Institute, Vitznau, Switzerland*
[2] *ZHAW School of Management and Law, Institute of Business Information Technology, Winterthur, Switzerland*
[3] *ZHAW School of Health Sciences, Institute of Occupational Therapy, Winterthur, Switzerland*
[4] *Faculty of Medicine, University of Zurich, Zurich, Switzerland*
[5] *Division of Vascular Neurology and Neurorehabilitation, Department of Neurology and Clinical Neuroscience Center, University of Zurich and University Hospital Zurich, Zurich, Switzerland*
[6] *cereneo, Center for Neurology and Rehabilitation, Vitznau, Switzerland*
[7] *ZHAW School of Engineering, Centre for Artificial Intelligence, Winterthur, Switzerland*
[8] *Neurocore Lab, Lake Lucerne Institute, Vitznau, Switzerland*
[†] *shared first-authorship*
[‡] *shared last-authorship*
[*] *corresponding author*

Correspondence*:
Tim Unger
tim.unger@llui.org

SUPPLEMENTARY RESULTS: LEAVE-ONE-SUBJECT-OUT (LOSO) WITH SMOTE

Rationale and Setup

To complement the stratified 3-fold cross-validation reported in the main paper, we conducted an additional leave-one-subject-out (LOSO) analysis. In each LOSO fold, all data from one subject were held out for testing, while the remaining subjects formed the training set. To handle class imbalance, we applied SMOTE only to the training data within each fold. The test set (held-out subject) remained untouched to avoid information leakage.

Results

Table 1 summarizes the inter-participant results for LOSO using SMOTE. The performance patterns confirm the findings reported in the main paper: mild compensations are generally classified more accurately and with higher recall than moderate compensations. The LOSO analysis highlights the same trend, while also showing greater variability for moderate cases, which can be attributed to the limited number of patients exhibiting sufficient moderate compensations. These supplementary results thus confirm that the small number of moderate cases limits generalization, whereas the larger pool of mild cases enables more consistent performance across participants. In particular, the OMC Moderate results degrade under LOSO (Figure 2), underscoring the challenges of generalizing from few moderate examples.

Error Analysis via Confusion Matrices

Figure 2 compares the inter-participant confusion matrices from the main paper (stratified 3-fold CV) with the new LOSO results. In both analyses, mild compensations consistently outperformed moderate compensations in inter-participant settings. This pattern is in line with the aggregate bar plots (Fig. 3) and supports the interpretation that the small number of patients with moderate compensations increases fold-to-fold variability. It should also be noted that the table reports results trained across all participants, whereas the bar plot highlights the five patients with sufficient moderate trials. This confirms that OMC Moderate and Hybrid Close Moderate do not generalize well across participants, reflecting the strong inter-individual variability of compensatory patterns.

Notes on Class Imbalance

In the main pipeline, undersampling was adopted for interpretability and stable accuracy. Here, LOSO with SMOTE demonstrates that the central findings are robust to alternative imbalance handling. Both approaches yielded consistent results, confirming that the conclusions are not sensitive to the specific method chosen to address class imbalance.

Custom Features Method			Standalone	Hybrid	OMC (A/R/P)
**Comp.**	**Cam.**	**Metric**	**Inter**	**Inter**	**Inter**
mild	center	balanced accuracy	0.43 (± 0.22)	0.49 (± 0.24)	0.68 (± 0.19)<br>0.62 (± 0.20)<br>0.66 (± 0.18)
		recall	0.41 (± 0.23)	0.47 (± 0.26)	
		precision	0.46 (± 0.20)	0.50 (± 0.21)	
	far	balanced accuracy	0.50 (± 0.14)	0.55 (± 0.16)	
		recall	0.47 (± 0.18)	0.52 (± 0.20)	
		precision	0.57 (± 0.12)	0.61 (± 0.16)	
	close	balanced accuracy	0.57 (± 0.22)	0.61 (± 0.18)	
		recall	0.55 (± 0.25)	0.57 (± 0.22)	
		precision	0.60 (± 0.20)	0.59 (± 0.19)	
moderate	center	balanced accuracy	0.66 (± 0.25)	0.69 (± 0.25)	0.66 (± 0.25)<br>0.47 (± 0.05)<br>0.46 (± 0.13)
		recall	0.49 (± 0.14)	0.51 (± 0.16)	
		precision	0.51 (± 0.15)	0.52 (± 0.16)	
	far	balanced accuracy	0.65 (± 0.26)	0.74 (± 0.18)	
		recall	0.49 (± 0.18)	0.56 (± 0.14)	
		precision	0.51 (± 0.13)	0.53 (± 0.14)	
	close	balanced accuracy	0.64 (± 0.24)	0.68 (± 0.25)	
		recall	0.47 (± 0.09)	0.50 (± 0.12)	
		precision	0.50 (± 0.12)	0.51 (± 0.15)	

Table 1. Test Balanced Accuracy (A), Recall (R), and Precision (P) for inter-participant classification using Standalone, Hybrid, and OMC features. Rows are grouped by compensation severity and camera angle. Results are shown as mean (± SD) for inter-participant evaluation.

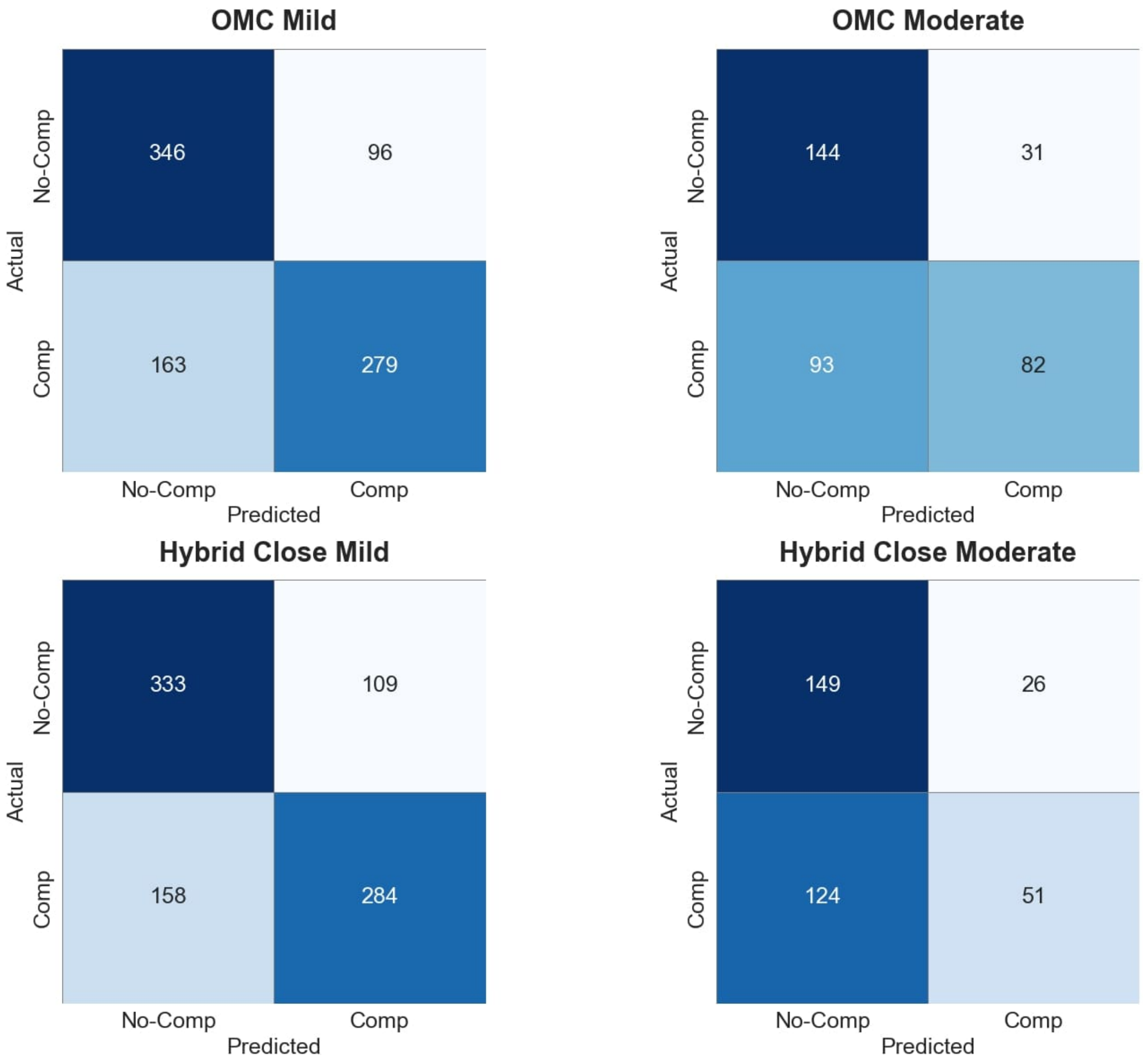

Figure 1. Confusion matrices from the main paper (3-fold stratified CV). Moderate compensations were recognized slightly better in this setup.

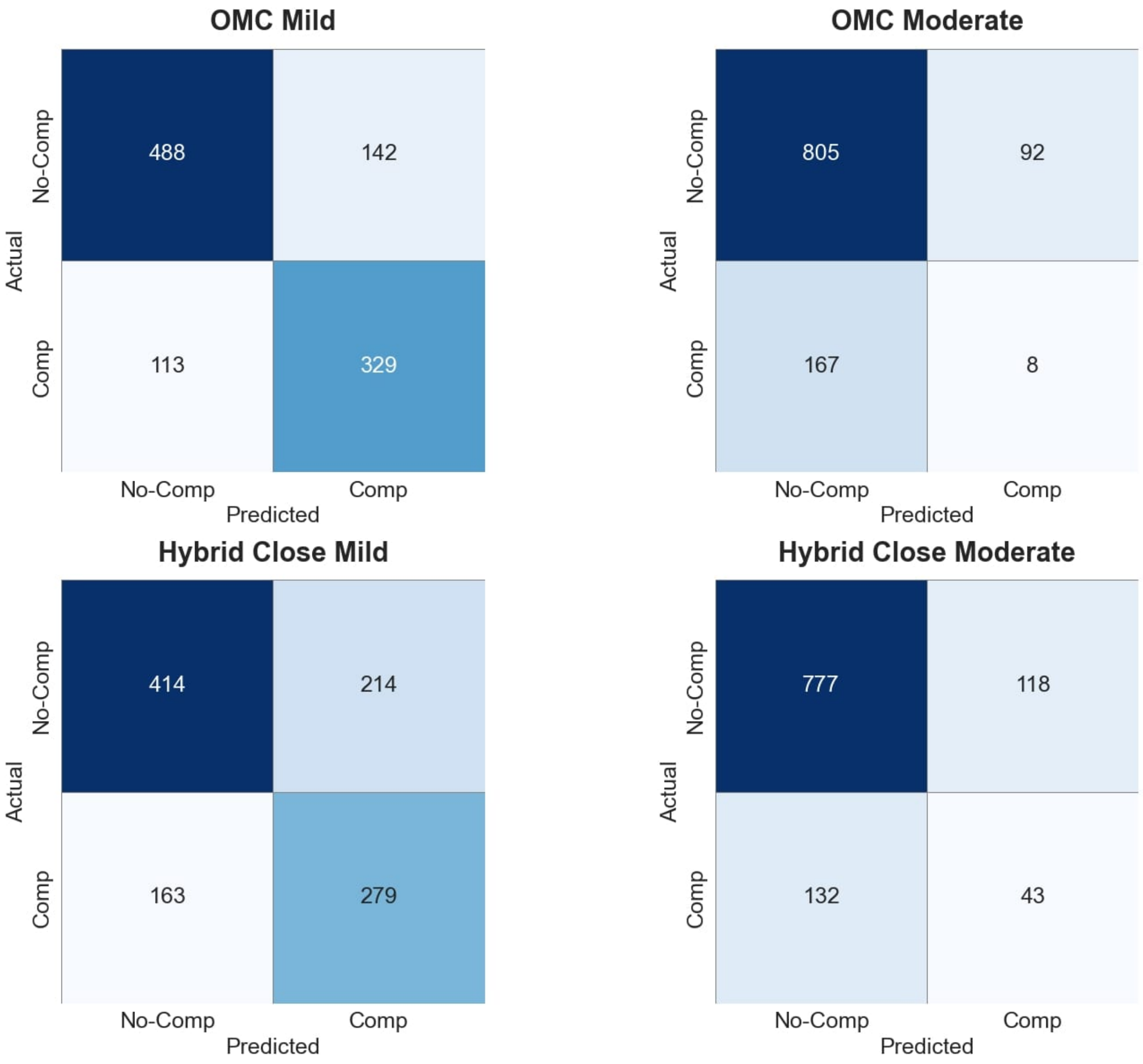

Figure 2. Confusion matrices from the LOSO analysis with SMOTE. Mild compensations are now recognized more reliably, aligning with the supplementary analysis.

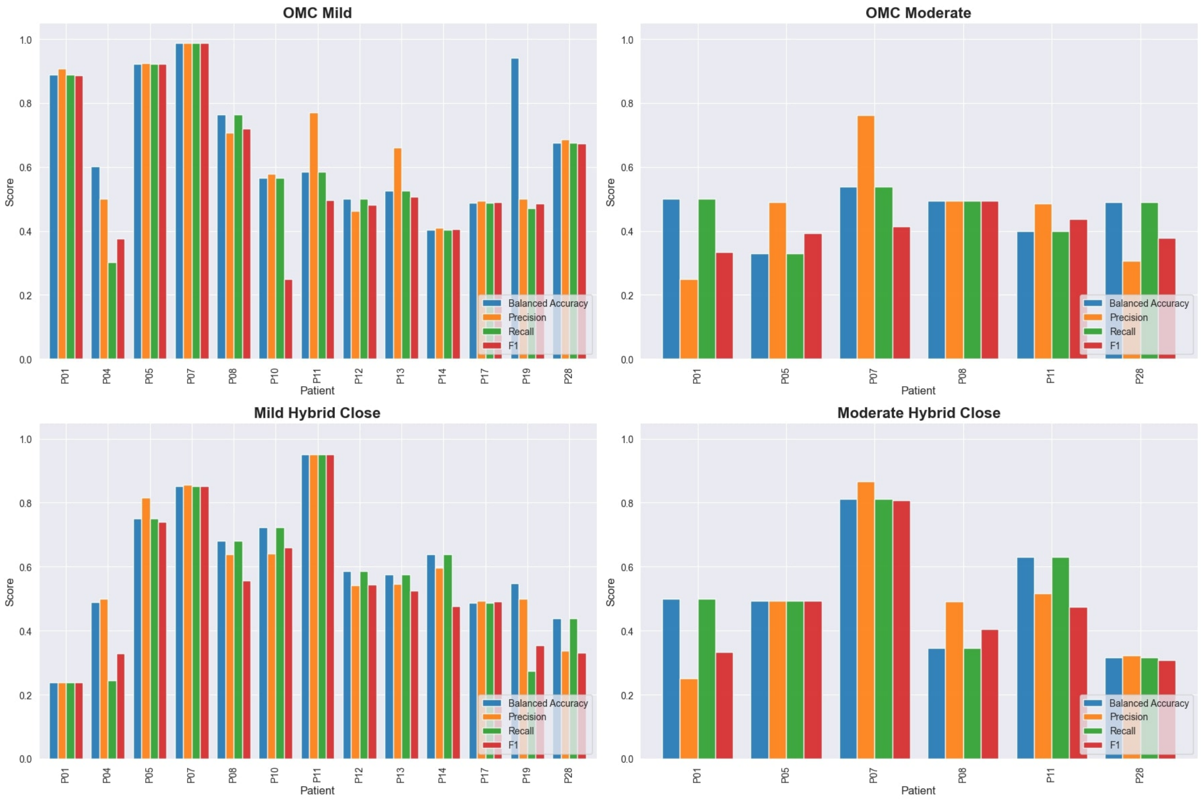

Figure 3. Aggregate inter-participant LOSO performance (bar plot). Consistent with the confusion matrices, mild compensations generalize more reliably than moderate compensations. *Note: For moderate compensations, only the five patients with at least five valid trials are included, to ensure stable performance estimates.*
