# Supplementary figures and images for: Using deep learning to detect upper limb compensation in individuals post-stroke using consumer-grade webcams—A feasibility study
Source: Front Med (Lausanne). 2025 Nov 14;12:1645369. doi: 10.3389/fmed.2025.1645369 (PMC12660197; doi:10.3389/fmed.2025.1645369)

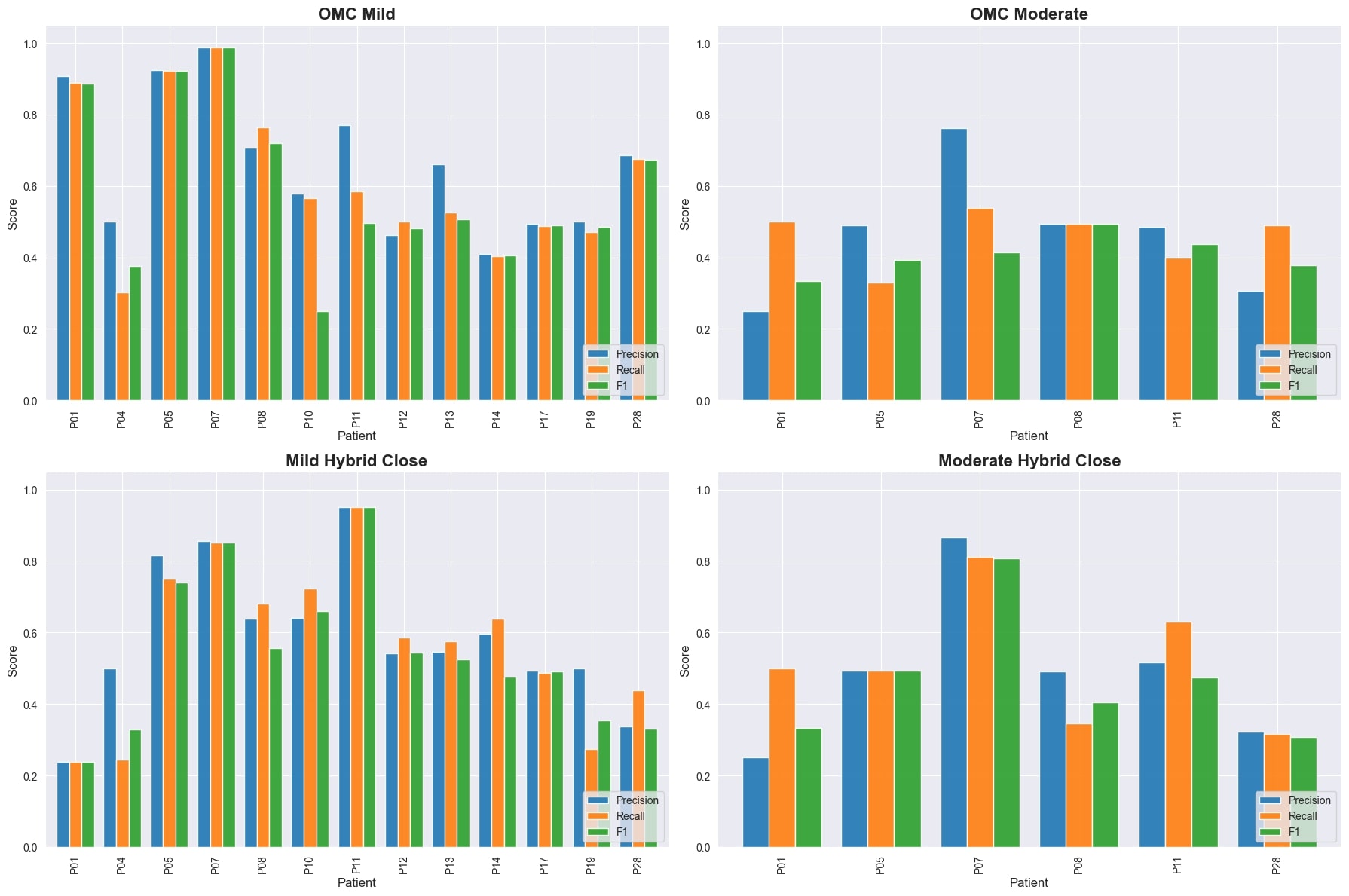

Supplement: Supplementary file 2 [file Image_1.jpeg]

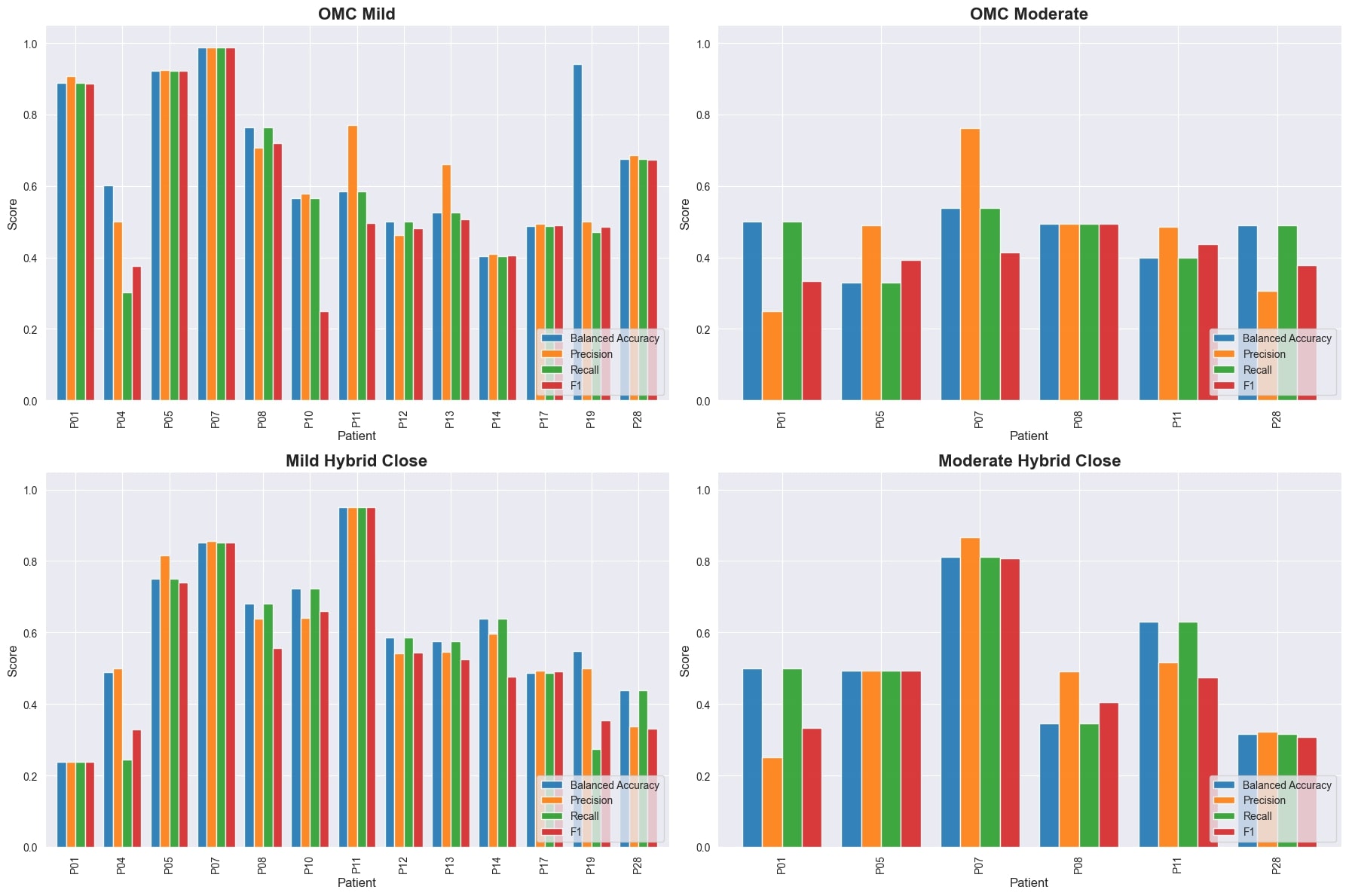

Supplement: Supplementary file 3 [file Image_2.jpeg]

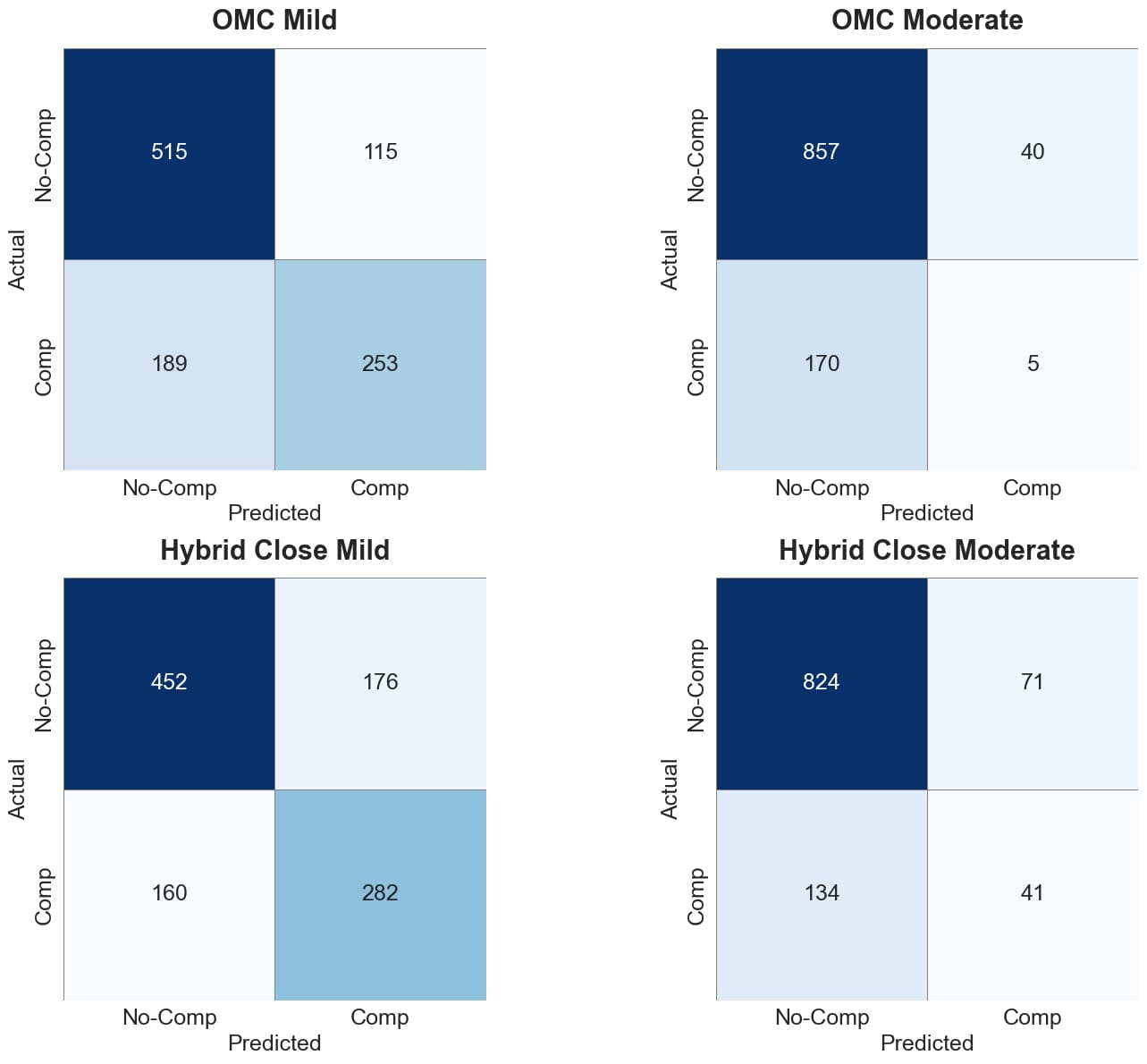

Supplement: Supplementary file 4 [file Image_3.jpeg]

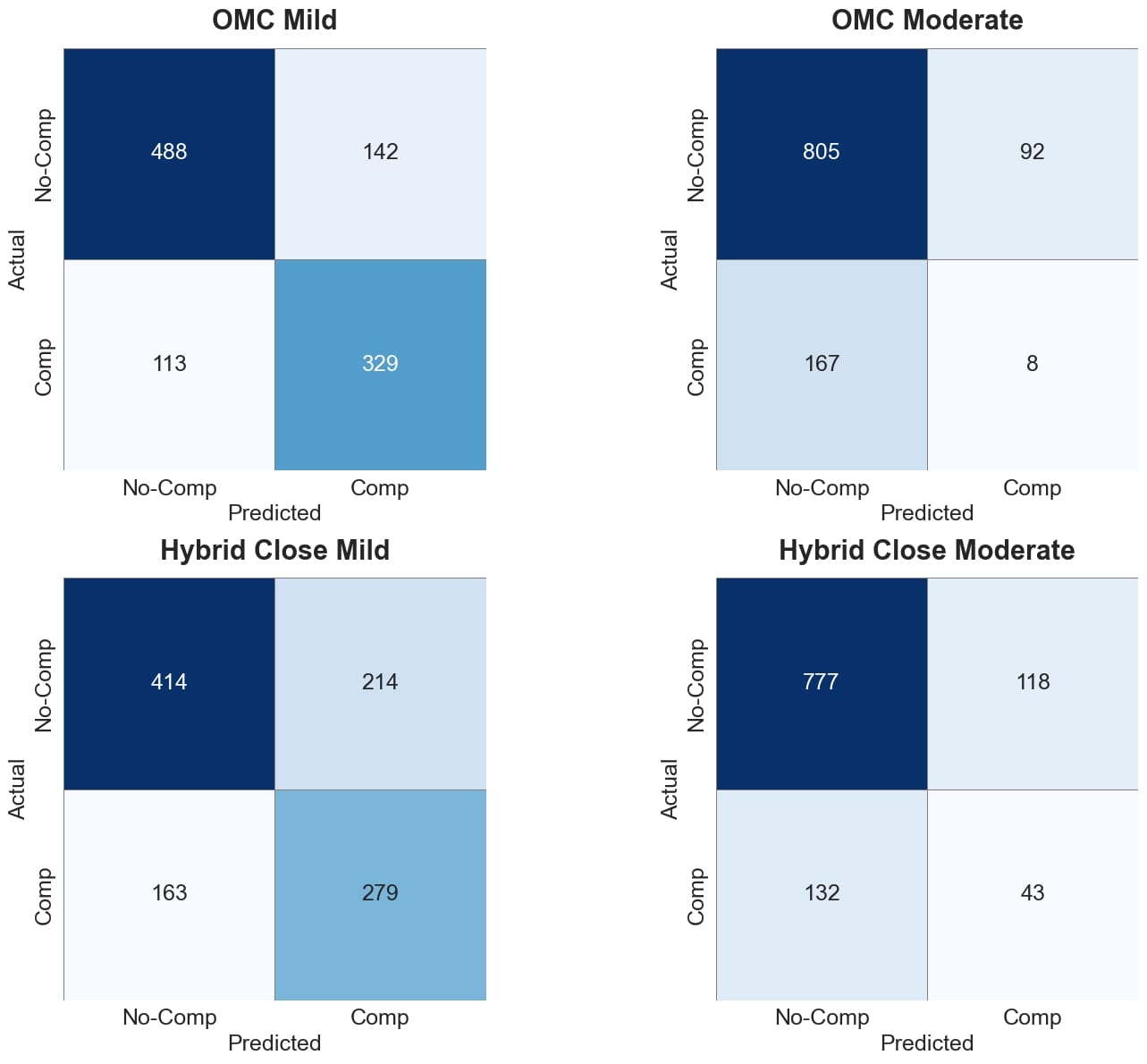

Supplement: Supplementary file 5 [file Image_4.jpeg]

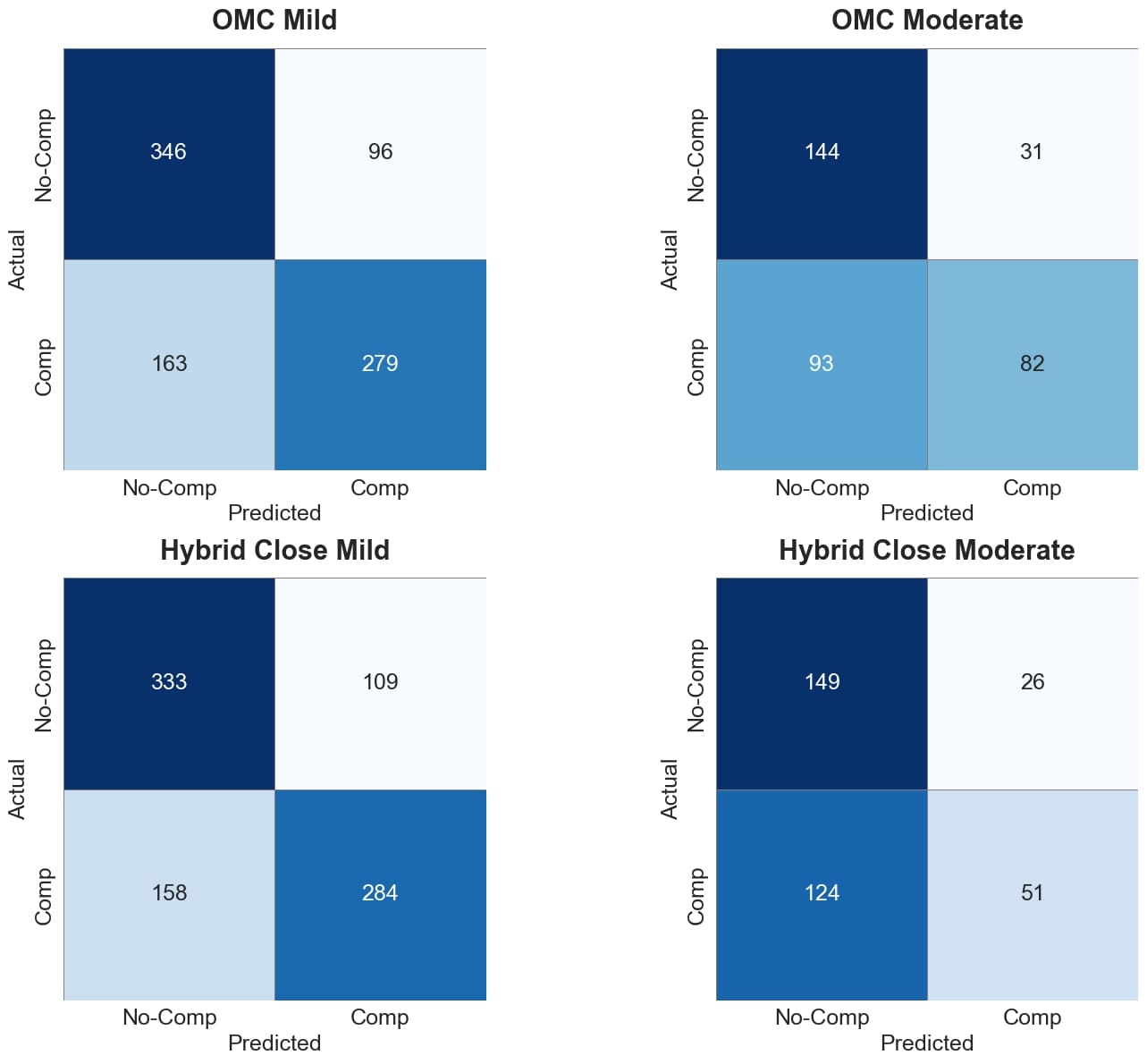

Supplement: Supplementary file 6 [file Image_5.jpeg]
